# Neurological Outcomes in Late Preterm Infants: An Updated Review of Recent Research and Clinical Insights

**DOI:** 10.3390/diagnostics15121514

**Published:** 2025-06-14

**Authors:** Andreea-Ioana Necula, Roxana Stoiciu, Razvan Radulescu Botica, Cristiana-Elena Durdu, Roxana Bohiltea

**Affiliations:** 1 Department of Neonatology, MedLife, 013695 Bucharest, Romania; andreea.necula@s.utm.ro; 2 Department of Obstetrics and Gynecology, MedLife, 013695 Bucharest, Romania; rrbotica@yahoo.com; 3 Department of Obstetrics and Gynecology, Filantropia Clinical Hospital, 011132 Bucharest, Romania; dr.bohiltea@gmail.com; 4 Doctoral School, “Carol Davila” University of Medicine and Pharmacy, 020956 Bucharest, Romania; 5 Department of Obstetrics and Gynecology, “Carol Davila” University of Medicine and Pharmacy, 020956 Bucharest, Romania

**Keywords:** preterm, neurological follow-up, neurodevelopment, low birth weight, neurological assessment, preterm outcomes

## Abstract

Research on late preterm infants is limited compared with extremely low birth weight infants, despite their vulnerability to brain injury. Early intervention is crucial, as these infants often face higher risks of cerebral palsy and developmental delays. This review examines methods to predict neurological outcomes and evaluates standard care protocols for neurologically affected late preterm infants. It also explores the potential for developing a comprehensive care bundle that integrates family involvement and delineates the responsibilities for continuous developmental monitoring. A total of 21 studies, primarily cohort studies, were included. This review synthesizes recent research on neurological development in late preterm infants, highlighting key markers and methods to improve neurological monitoring and long-term outcomes. Late preterm infants are at an increased risk for neurodevelopmental impairments, such as cerebral palsy and cognitive delays, particularly when growth restrictions or low birth weight are present. Early interventions, including specialized neurological assessments and targeted rehabilitation, show potential for improving these outcomes. Late preterm infants face increased neurodevelopmental risks despite low perinatal mortality. Early identification, standardized assessments, and targeted follow-up are essential. Emerging interventions show promise, but further research and equitable care access are needed to improve long-term outcomes.

## 1. Introduction

Extensive research on the neurodevelopmental outcomes of extremely low birth weight infants exists, but data on late preterm infants are limited. Given the brain’s vulnerability during this critical developmental stage, monitoring their neurological outcomes is essential.

Late preterm infants are at an increased risk of brain injury, as the last 6 weeks of gestation are crucial for brain growth and maturation. Early interventions may benefit these infants, especially those with neurological injury. Given the rising number of late preterm births, understanding and addressing their risks is crucial due to the potential economic and educational impact [1].

Late preterm infants are at an increased risk of respiratory infections, recurrent wheezing, asthma, and impaired lung function. This highlights the importance of a structured respiratory follow-up for these children [2]. Therefore, comprehensive and systematic monitoring from infancy through adulthood plays a critical role in supporting optimal neurological development. By ensuring early identification and timely intervention for potential complications, these monitoring strategies contribute to improved long-term health outcomes, supporting both respiratory and neurodevelopmental stability in this vulnerable population.

A study from 2019 highlights that late preterm infants often perform worse than full-term infants in cognitive and educational outcomes, with even small differences having significant implications. Key questions remain about identifying high-risk groups, the nature of developmental delays, and the role of early interventions [3]. A lower GA is associated with an increased risk of cerebral palsy (CP) and developmental delay/intellectual disability (DD/ID), including among infants born between 34 and 36 weeks [4].

A 2020 study tracked the development of general movements (GMs) in four infants from 3 weeks old until 4–5 years old. Initially, some infants had cramped–synchronized (CS) movements, which evolved into coordinated GMs. By term equivalent age, GMs improved, and by 24 months, all were neurologically normal with typical developmental scores [5]. This represents a highly valuable tool that is easily accessible, offering significant potential for widespread use and application.

This review seeks to address the following questions: how can we predict neurological outcomes, what standard care methods should be implemented for neurologically affected late preterm infants, can a comprehensive care bundle be developed to include family involvement, and who should be responsible for the ongoing monitoring of the child at each developmental stage to ensure age-appropriate progression?

## 2. Materials and Methods

In this review, we sought to analyze recent findings related to neurological development in late preterm infants, in contrast to their full-term peers. Specifically, we aimed to identify key markers that should be monitored, according to the latest studies, to enhance the quality of life for these infants. Articles published from 2023 onward were selected based on their relevance as assessed by the authors. Two reviewers (R.S. and C.E.D.) independently screened the studies for eligibility on the basis of the inclusion criteria, utilizing titles and abstracts; any disagreements were resolved by a third reviewer (A.I.N.). A second round of inclusion was conducted through full-text screening. The selection criteria encompassed late preterm infants, neurological assessments, neurological outcomes, and various methods to improve outcomes. Data extracted from each study included citation details, study design, country of origin, and specific outcomes. The accuracy and completeness of the extracted data were verified by A.I.N. Discrepancies were resolved through discussion, and primary studies were consulted as needed.

Data sources included PubMed, Cochrane Library, and Web of Science. The search strategy involved the use of the following keywords: preterm, neurological follow-up, neurodevelopment, low birth weight, neurological assessment, pediatric neurology, and preterm outcomes.

**Figure 1:**
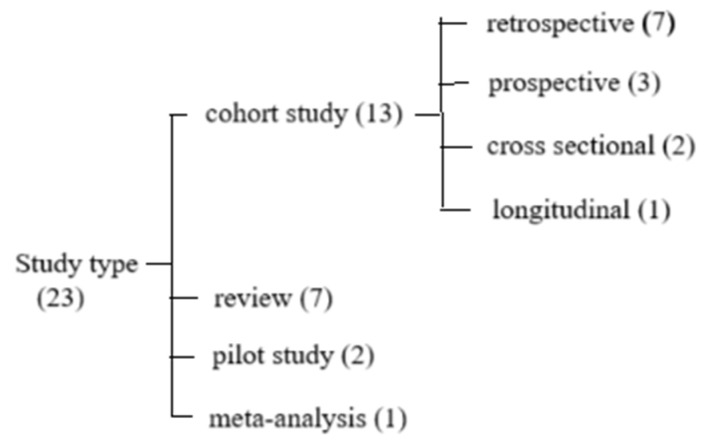
Study flow diagram—classification of study types included.

A total of 23 studies (Figure 1) met the inclusion criteria and were included in this review. The majority of these studies were cohort studies, which are well suited to investigate the long-term neurological outcomes of late preterm infants and to track the effectiveness of various interventions aimed at improving their development.

**Figure 2:**
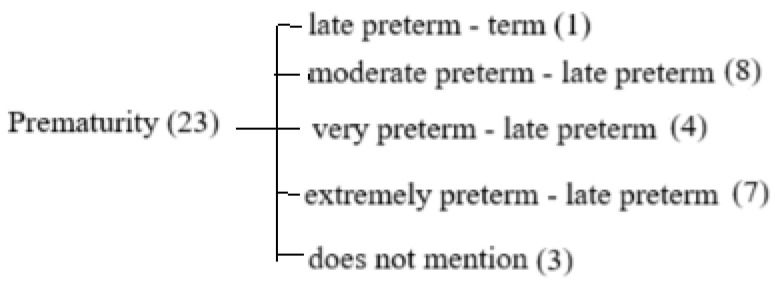
Study flow diagram—classification of prematurity.

Among the 23 studies (Figure 2) included in this review, only 1 (Triggs, T., et al., 2024 [6]) specifically examined late preterm infants in comparison with term infants. Eight studies (Paulsen H., et al., 2023 [7]; Toma A.I., et al., 2024 [8]; Mitha A., et al., 2023 [9], Ericson J., et al., 2023 [10]; Assar E.H., et al. 2024 [11]; Toma A.I., et al., 2024 [12]; Merino-Andres J., et al., 2024 [13]; Toma A.I. et al., 2024 [14]) focused on moderate to late preterm infants, four (Cote-Corriveau G., et al., 2023 [15]; Inder TE., et al., 2023 [16]; Zhang C., et al., 2024 [17]; Lubian-Gutierrez M., et al., 2024 [18]) included very to late preterm infants, and seven (Gonzales-Moreira E., et al., 2023 [19]; Bobba P.S., et al., 2023 [20]; Silveira R.C., et al., 2023 [21]; Song IG, 2023 [22]; Wolfsberger C.H., et al., 2023 [23]; Molloy E.J., et al., 2024 [24]) addressed extremely to late preterm infants. For the purposes of this analysis, only the data specifically related to late preterm infants were extracted and included in this review. Three studies did not specify the degree of prematurity; two (Toma A.I., 2023 [25]; Zhou M., et al., 2024 [26]) used only the general term “preterm” without indicating gestational age (GA) in weeks, and one study (Snir A., et al., 2024 [27]) classified infants using generalized GA ranges of <34 weeks and <37 weeks.

Neurological and motor functions of the infants were evaluated using the Hammersmith Infant Neurological Infant Examination (HINE), Prechtl General Movements Assessment (GMA), and Neonatal Behavioral Neurological Assessment (NBNA).

A publication by Paulsen et al. (2023) [7] claimed that the HINE consists of 3 components: neurological examination, motor milestone observation, and behavioral state. The neurological section comprises 26 items assessing cranial nerves, posture, movements, tone, and reflexes, each scored from 0 to 3 in 0.5-point increments (maximum score: 78). Scores at or below the 10th percentile are considered suboptimal. Valid reference data are available for term infants at 3–8, 12, and 18 months, and the tool has demonstrated strong validity in both high- and low-risk populations [7].

A study from Toma et al. (2024) [8] stated that the general movements (GM) pattern evolves during the first few months of life. At term, writhing movements are typically observed as the normal GM pattern. By the end of the second month and the beginning of the third, these are gradually replaced by fidgety movements, which become the predominant normal pattern. Fidgety movements can be observed and recorded up to around 5 months of corrected age (approximately 20 weeks), after which they are replaced by typical voluntary movements. Two GM patterns are strongly linked to motor impairment or CP: cramped–synchronized movements near term and absent fidgety movements at 3–4 months of corrected age. Cramped–synchronized movements involve rigid, simultaneous contractions of the limb and trunk muscles, lacking normal fluidity. A poor repertoire pattern is characterized by monotonous, repetitive movements with limited complexity and variability across body parts [8].

Work published by Zhang et al. (2024) [17] revealed that the NBNA evaluates neonatal behavior and facilitates early detection of mild brain injury. It covers 5 domains (behavioral capacity, passive and active muscle tone, primitive reflexes, and general assessment) across 20 items on a 40-point scale. Recent studies support its utility in diagnosing brain injury in preterm infants and animal models [17].

One study from Toma et al. (2024) [8] suggested implementing an intervention involved a structured 12-week physical therapy program, utilizing either Incipient Bobath Therapy or Vojta Therapy, as determined by the treating therapist. Sessions were conducted at a minimum frequency of three times per week. Parental involvement was a key component, with caregivers being instructed in selected therapeutic techniques to support daily at-home implementation [8]. Further investigation is warranted to evaluate interventions that may enhance long-term neurological outcomes in late preterm infants.

## 3. Results

During the literature review, we determined that we needed to analyze the following categories involved in the neurological outcomes of late preterm infants: birthweight and growth restriction, nutrition, cerebral oxygenation, hyperbilirubinemia, neurological assessment, and outcomes (Table 1).

### 3.1. Birthweight and Growth Restriction

Late preterm infants face higher perinatal mortality and neonatal morbidity risks than term infants, though the absolute incidence remains low. While perinatal mortality rates remained stable, severe neurological and other morbidities increased in the later study period, possibly due to changes in reporting or earlier obstetric interventions for perceived risks [6]. Fetuses suspected of fetal growth restriction (FGR) but born with a normal birth weight had a higher risk of neurological disorders, including movement impairments and cerebral palsy. Preterm delivery at 34–37 weeks was significantly higher in cases with a misdiagnosis of FGR (36.4%) compared with those without misdiagnosis (6.6%). Misdiagnosed FGR may contribute to adverse outcomes, as iatrogenic early deliveries are linked to neurodevelopmental impairments. Cesarean delivery also emerged as an independent risk factor for pediatric neurological hospitalizations. Even after adjusting for confounders, infants classified as appropriate for gestational age (AGA) may have experienced in utero growth restriction, leading to outcomes like those with lower birth weights [27]. Male sex and low birth weight were also over-represented in moderate to late preterm infants, with low birth weight further increasing impairment risks [9].

### 3.2. Nutrition

Extrauterine growth restriction (EUGR) definitions and their potential link to adverse neurodevelopmental outcomes in extremely to late preterm infants should be taken into consideration. However, inconsistencies in EUGR definitions and neurodevelopmental assessments hinder definitive conclusions, underscoring the need for standardized criteria [28]. Consistent criteria for EUGR and neurodevelopmental assessments are crucial for ensuring consistency in outcome evaluations.

As highlighted in this review, the period from 24 to 52 weeks post-conception is critical for neural development, with the gut–brain axis and microbiome playing key roles. Premature infants, with immature gut and blood–brain barriers, face heightened neurodevelopmental risks. Human milk, the optimal nutrition for preterm infants, provides essential macronutrients, micronutrients, neuropeptides, and neurohormones that support brain development. Breastfeeding enhances maternal–infant bonding and is linked to improved white matter microstructure, larger brain volumes, better language skills, and superior cognitive outcomes into later years, particularly in males. However, the impact of donor milk and pasteurization on neurodevelopment remains uncertain, emphasizing the need for individualized nutrition strategies [21]. Whereas, in a cohort of moderately preterm infants, no significant differences in neurological, growth, or health outcomes were found up to 6 years between those fed exclusive breast milk and fortified breast milk and/or formula. Human milk fortification did not enhance growth, and no differences were observed in illness, well-being, or developmental outcomes [10]. Evidence for nutrient and probiotic supplementation remains inconclusive for extremely to late preterm infants [22]. These findings underscore the complexity of neonatal nutrition and the need for further research to optimize preterm infant neurodevelopment.

### 3.3. Cerebral Oxygenation

Neonates with adverse long-term outcomes exhibited lower GA, reduced cerebral regional oxygen saturation (crSO_2_), and increased cerebral fractional tissue oxygen extraction (cFTOE) during the fetal-to-neonatal transition, despite no significant differences in peripheral arterial oxygen saturation (SpO_2_), heart rate, or fraction of inspired oxygen (FiO_2_), except for a brief variation at 1 min. These findings on extremely to late preterm infants suggest that impaired cerebral oxygenation during transition may contribute to adverse long-term outcomes. Short-term morbidities, including periventricular leukomalacia (PVL), bronchopulmonary dysplasia (BPD), intestinal perforation, and sepsis, were significantly more prevalent in the adverse outcome group. Long-term outcomes, assessed at 24 months of corrected age, revealed that three neonates had severe cognitive impairment precluding Bayley Scales of Infant and Toddler Development Second/Third Edition (BSID II/III) testing, while two were diagnosed with cerebral palsy [23]. These findings emphasize the need to monitor cerebral oxygenation as a potential predictor of long-term developmental outcomes in preterm and high-risk infants.

Another study focused on very to late preterm infants found that while Functional Diffuse Near-Infrared Spectroscopy and Diffuse Correlation Spectroscopy (FDNIRS-DCS) parameters were not linked to neurological examination at TEA, greater increases in the index of microvascular cerebral blood flow (CBFi) and the index of cerebral oxygen metabolism (CMRO2i) correlated with higher GA [15]. FDNIRS-DCS may serve as a complementary tool to traditional assessments by evaluating cerebral blood flow and oxygen metabolism in preterm infants.

The two studies presented here underscore the potential of a complementary tool in the assessment of newborns. Future research should aim to extend its application to late preterm infants, identifying this group as a distinct target population. Within this cohort, it will be essential to stratify infants, with particular attention to those who are critically ill, to facilitate more precise and individualized care approaches.

### 3.4. Hyperbilirubinemia

A cross-sectional study found that moderate to late preterm infants had significantly lower hemoglobin levels than full-term infants, with no differences in white blood cells (WBCs), platelets, reticulocytes, red blood cell (RBC) counts, or bilirubin levels. Jaundice in preterm infants was primarily due to prematurity (70.3%) and sepsis (21.6%) (*p* < 0.001). Kernicterus rates did not differ significantly (10.7% vs. 19%, *p* = 0.22). Adverse outcomes, including mortality (32.4%) and developmental delay (29.7%), were significantly higher in preterm infants. The original article does not provide a clear distinction between bilirubin encephalopathy, BIND, and kernicterus. Further research is warranted to establish more precise clinical and pathological criteria differentiating these bilirubin-associated neurological conditions, as that study does not adequately address this gap in the literature. Specific developmental concerns included impaired head control (8.1%), social smile (8.1%), response to sound (18.9%), and eye contact (18.9%), as well as abnormal movements (8.1%), hypotonia (5.4%), hypertonia (2.7%), and absent (5.4%) or exaggerated reflexes (2.7%) [11].

Elevated bilirubin levels may contribute to neurodevelopmental disorders in full-term infants, particularly within the first year of life. However, no clear impact was observed in moderate to late preterm infants [13]. Additional research is necessary to assess the impact of hyperbilirubinemia on neurodevelopment in preterm infants.

### 3.5. Neurological Assessment

Preterm birth is associated with significant neurodevelopmental challenges, with multiple studies highlighting the risks of motor and cognitive impairments. A prospective cohort study found that preterm infants scored lower on neurological and motor assessments at 3 to 7 months, with a mean difference of −1.5 on the Hammersmith Infant Neurological Examination (HINE) compared with term AGA infants. Additionally, a significant proportion of preterm infants scored below the 10th percentile on the HINE, and a higher odds ratio for scoring below the cut-off on the Ages and Stages Questionnaires second edition (ASQ-2) gross motor assessment was observed. By 2 years, preterm children exhibited poorer gross motor function, underscoring the increased risk of neurological deficits in infancy and motor impairments at 2 years [7].

Early intervention has shown promise in improving neuro-motor outcomes in preterm infants. Infants exhibiting a cramped–synchronized GM pattern at term-equivalent age benefited significantly from early intervention, with most developing normal fidgety movements. Since these movements are strongly linked to a low risk of cerebral palsy, these findings suggest a favorable prognosis, emphasizing the importance of therapeutic intervention and family involvement [8,25]. It is important to acknowledge that this was a monocentric pilot study, and additional data are required to draw definitive conclusions, as reliance on this dataset alone is insufficient.

Structural brain abnormalities correlate with atypical GM patterns in preterm neonates. The CS pattern was associated with lateral ventricular enlargement, indicative of white matter injury, and reduced basal ganglia diameter. The poor repertoire (PR) pattern correlated with increased sinocortical width and reduced pons diameter, suggesting that distinct structural alterations contribute to GM dysfunction. These findings underscore the role of subplate neurons, basal ganglia, and cortical structures in motor impairments [12]. Neurodevelopmental assessments at 40 weeks corrected GA using the Neonatal Behavioral Neurological Assessment (NBNA) demonstrated, in a retrospective cohort study, significantly lower scores in preterm infants, with strong positive correlations between NBNA scores and mean kurtosis (MK) values in the genu of the corpus callosum (GCC), thalamus (TH), and radial kurtosis (RK) of the cerebellum, as well as brain parenchymal fraction (BPF) [17].

Katona’s neurohabilitation method, combining diagnostic and therapeutic elements, has demonstrated, in a retrospective cohort study, significant benefits when initiated before 3 months. Preterm infants who underwent this intervention exhibited superior outcomes at 3 years, highlighting the importance of early and intensive rehabilitation. Additionally, sepsis and corpus callosum and lateral ventricle volumes at 3–4 months emerged as key predictors of 3-year outcomes [19].

A prospective cohort study revealed that long-term neurodevelopmental consequences persist into childhood. School-aged preterm children with smaller corpus callosum measurements, particularly in the posterior region, showed deficits in cognition and motor function, likely due to impaired sensory–motor integration [18].

Preterm brain injuries significantly impact cognitive, motor, and developmental outcomes, with diffuse white matter injury (WMI) being the most prevalent. Cystic WMI, often resulting from hypoxic–ischemic events, is exacerbated by inflammation. Emerging therapeutic options, including anti-inflammatory agents such as etanercept and medications like erythropoietin, melatonin, and caffeine citrate, show potential but require further validation. Non-pharmacological interventions, such as skin-to-skin care and proper nutrition, also play a vital role in optimizing outcomes. A comprehensive care model integrating strategies to prevent acute brain injury and enhance neuroplasticity is essential. Despite advances in neonatal care, the incidence of CP remains high. While antenatal treatments such as corticosteroids and magnesium sulfate offer benefits, their long-term effectiveness remains uncertain. Postnatal treatments like therapeutic hypothermia yield mixed results in preterm infants, and ongoing research into pluripotent stem cell therapies continues [24].

The severity of intraventricular hemorrhage (IVH) in preterm infants correlates with neurodevelopmental impairment, with even mild IVH increasing the risk of cognitive, motor, and sensory deficits. Early detection and timely intervention are critical to mitigating long-term consequences, necessitating continuous monitoring and a multidisciplinary approach throughout early development [26]. A magnetic resonance imaging (MRI) retrospective cohort study revealed that preterm infants delivered via C-section exhibit delayed white matter maturation, independent of GA and other factors, reinforcing the need for close neurological surveillance [20].

### 3.6. Outcomes

Preterm births were more common among mothers with young age, obesity, smoking, diabetes, hypertension, lower education, and a psychiatric history. Over a median follow-up of 13.1 years, 75,311 children (47.8 per 10,000 person-years) were diagnosed with neurodevelopmental impairments, most commonly cognitive (17.0 per 10,000), hearing (12.6 per 10,000), and visual (12.2 per 10,000) impairments. Children born moderately or late preterm had higher risks of neurodevelopmental impairments than full-term children. Risk declined with increasing GA, with the most significant reduction in impairment seen if children born at 37–38 weeks were instead born at 39–40 weeks. Sibling analysis confirmed the findings, except for epileptic and hearing impairments. Risk patterns were similar between spontaneous and induced labor, with some differences in motor and cognitive outcomes. Late preterm infants (34–36 weeks) showed increased risks across several neurodevelopmental domains by age 16. Compared with full-term infants, they had a 30% higher risk for any impairment (hazard ratio: 1.30), with elevated risks for motor (hazard ratio: 1.90), visual (hazard ratio: 1.42), cognitive (hazard ratio: 1.31), epileptic (hazard ratio: 1.23), and hearing (hazard ratio: 1.16) outcomes. The risk of severe or major impairments was also increased (hazard ratio: 1.55) [9]. Aberrant gross and fine motor outcomes in late preterm infants at 12–24 months of corrected age demonstrate a significant association with abnormal findings on head ultrasound examinations conducted at term-equivalent age (TEA). These abnormalities suggest the involvement of both white matter, indicated by an increased midbody distance, and grey matter, reflected in a reduced basal ganglia diameter, decreased cortical depth, and an immature gyration pattern [14].

Early brain injury recognition and rehabilitative strategies during and after Neonatal Intensive Care Unit (NICU) care may aid neurorestoration. Improvements in nutrition, parental support, and engagement are also suggested to enhance neurodevelopmental outcomes [16]. Additional research is necessary to explore the neurobiological effects of sensory environments, stress, and sleep in preterm infants.

Moderate and late preterm infants typically present with less severe clinical complications than those born very preterm; nevertheless, they remain at increased risk of neurodevelopmental impairments (NDI) compared with term infants. Early recombinant human erythropoietin (rhEPO) may aid cognitive development in very preterm infants but shows no impact on motor or sensory outcomes [22].

## 4. Discussion

A limitation of this review is that none of the included studies focused exclusively on late preterm infants. Given the scope of this review, it sought to elucidate the heightened susceptibility of late preterm infants to brain injury in comparison with their term peers. Among the studies analyzed, only one directly compared late preterm and term infants. Several examined moderate to late, very to late, or extremely to late preterm populations. A few did not clearly define GA. For this review, only data relevant to late preterm infants were extracted and analyzed.

We can predict neurological outcomes by taking into consideration critical factors such as prematurity, early iatrogenic delivery, and EUGR. However, regarding nutritional interventions, current studies have not demonstrated a clear improvement in neurological outcomes among late preterm infants receiving human milk. Similarly, the influence of hyperbilirubinemia and impaired cerebral oxygenation on long-term neurodevelopmental prognosis remains inconclusive. Thus, while these clinical parameters provide a basis for prediction, further research is needed to clarify their individual and combined effects on neurological development in this population.

Late preterm infants face increased neurodevelopmental risks despite low perinatal mortality rates [6]. Early deliveries, especially for suspected fetal growth restriction (FGR), may contribute. Even when misdiagnosed, infants with normal birth weights suspected of FGR show higher risks of movement disorders and cerebral palsy [27]. Male sex and low birth weight further elevate the risks, highlighting the need for early screening and individualized follow-up care [9]. Inconsistent definitions of extrauterine growth restriction (EUGR) and neurodevelopmental assessments complicate outcome evaluations [28]. A 2015 prospective cohort study confirms that infants born between 32 and 36 weeks’ gestation face twice the risk of neurodevelopmental disabilities by age 2 compared with their full-term counterparts. The study further identifies male sex and socioeconomic disadvantage as independent predictors of lower cognitive scores in these infants [29].

Neural development from 24 to 52 weeks post-conception is shaped by the gut–brain axis. Late preterm infants have immature gut and blood–brain barriers, increasing the risk and making nutrition vital. Human milk supports brain growth and is linked to better cognitive outcomes, especially in males [21], though the effects of donor milk and pasteurization remain unclear. Studies show no significant long-term neurological differences between infants fed breast milk alone vs. fortified milk or formula [10]. Fortification showed no growth benefit, and data on nutrient/probiotic supplementation are inconclusive [22]. Additional cohort studies are needed to further investigate the significance of neonatal hypoglycemia and its effects on neurodevelopment. The aim is to elucidate the impact of early-life low blood glucose levels on cognitive and motor development in children. In a recent review, neonatal hypoglycemia was associated with an increased risk of neurodevelopmental impairments, including cognitive delays and motor dysfunction [30].

Impaired cerebral oxygenation during the birth transition may lead to adverse outcomes, as infants with poorer prognosis showed lower GA, reduced crSO_2_, and increased cFTOE, despite a similar SpO_2_, heart rate, and FiO_2_. These infants also had higher rates of PVL, BPD, intestinal perforation, and sepsis, with some developing severe cognitive impairment or cerebral palsy by 24 months [23]. Emerging monitoring tools like FDNIRS-DCS may support brain assessment; although not directly linked to neurological scores at term-equivalent age, higher CBFi and CMRO2i values were associated with greater GA, indicating potential for evaluating neurodevelopmental status [15].

Certain pulmonary complications in late preterm infants may potentially be addressed through surfactant therapy, and further research is warranted to explore the additional challenges faced by this cohort. A 2024 study demonstrated that preterm infants born at less than 28 weeks GA experienced a reduction in BPD following less/minimally invasive surfactant administration/therapy (LISA/MIST) [31].

Preterm infants exhibit lower hemoglobin levels compared with full-term infants, primarily due to underdeveloped erythropoiesis. This condition generally improves with iron supplementation over time [32]. While high bilirubin levels correlate with neurodevelopmental disorders in term infants, effects in preterm infants remain unclear [13]. Furthermore, elevated bilirubin levels, which are prevalent in preterm infants, are associated with neurodevelopmental impairments and hearing deficits [33]. Jaundice, often linked to prematurity and sepsis, shows no significant difference in kernicterus rates. Adverse outcomes such as motor delays, abnormal reflexes, and sensory impairments are more frequently observed [11]. These conditions underscore the importance of careful monitoring and management in preterm infants to reduce long-term developmental risks.

Standardized care methods should be implemented for neurologically affected late preterm infants to ensure consistent and comprehensive assessment across developmental stages. Given the current variability in evaluation tools, a unified approach would facilitate early identification of deficits, enable timely interventions, and promote equitable care across clinical settings.

Preterm birth is strongly tied to motor and cognitive impairments, with lower neuro scores and higher deficits by age 2 [7]. Early intervention, especially in those with atypical GMs, may improve outcomes and lower cerebral palsy risk [8,25]. A 3-year follow-up revealed that infants who received the therapy demonstrated significantly higher scores on both the Mental Developmental Index (MDI) and the Psychomotor Developmental Index (PDI) compared with those who did not undergo the treatment [19]. Structural brain issues—ventricular enlargement and smaller basal ganglia—are linked to motor dysfunction, highlighting the role of subplate neurons and cortical structures [12]. Assessments at term-equivalent age exhibit strong correlations with brain microstructure [19] and are effective in predicting outcomes, demonstrating a significant association between preterm birth and motor/cognitive impairments, including cerebral palsy [34].

Early neurohabilitation, such as Katona’s method, shows benefits if started before 3 months, improving outcomes by age 3. Key predictors include sepsis and volumes of the corpus callosum and lateral ventricles [19]. Smaller corpus callosum size in school-aged children is linked to cognitive and motor deficits, reinforcing the importance of early intervention [18]. A 2017 study reported that 80% of infants exhibited abnormal MRI findings, including increased lateral ventricle volumes and reduced corpus callosum volume. Nevertheless, the therapy demonstrated potential in enhancing developmental outcomes [35].

Preterm brain injuries, particularly diffuse WMI, significantly impact neurodevelopment. Emerging therapeutic strategies, including anti-inflammatory agents, erythropoietin, melatonin, and caffeine citrate, show promise but require further validation. Non-pharmacological interventions, such as skin-to-skin care and optimal nutrition, remain critical components of care [24]. Early recognition of brain injury and interventions like recombinant human erythropoietin (rhEPO) may support cognitive development in very preterm infants. However, rhEPO does not appear to affect motor or sensory outcomes. Other strategies, such as improved nutrition and parental engagement, are essential for enhancing neurodevelopment [16,22].

Preclinical studies suggest that melatonin can reduce microglial activation, decrease proinflammatory mediators, and prevent oxidative damage in the developing brain. Additionally, melatonin’s ability to cross the placenta and its favorable safety profile make it a promising candidate for both antenatal and postnatal neuroprotection [36].

Maternal factors like young age, obesity, smoking, diabetes, hypertension, and lower education are linked to higher preterm birth rates, which, in turn, increase the risk of neurodevelopmental impairments, including cognitive, hearing, and visual issues. Addressing these risk factors could reduce the incidence of preterm births and improve long-term outcomes for children [9]. Some studies suggest that while these maternal factors contribute to the risk, they are part of a broader spectrum of influences, including genetic predispositions and environmental conditions. For instance, research indicates that maternal genetics account for approximately 22.8% of the variations in GA, and fetal genetics contribute around 12.7%. This underscores the notion that addressing maternal factors alone may not comprehensively mitigate the risk of preterm births and the associated neurodevelopmental issues [37].

Preterm infants, even those born moderately or late preterm, often show motor and cognitive impairments at 12–24 months of corrected age. Abnormalities in head ultrasound at term-equivalent age suggest both white and gray matter involvement. This emphasizes the need for early interventions to address these challenges [14]. Another relevant study demonstrates that abnormalities in brain microstructure and metabolism detected early in life are associated with adverse neurodevelopmental outcomes at 18 months of corrected age. Specifically, impairments in the maturation of brain microstructure and metabolism, particularly in the basal nuclei, were strongly linked to adverse outcomes across various developmental domains. This research emphasizes that brain abnormalities in preterm newborns extend beyond visible structural lesions, highlighting the importance of early screening [38].

The neurological outcomes of late preterm infants remain a critical area of concern, given their increased susceptibility to developmental challenges. In cases of pre-labor preterm rupture of membranes (PPROM), when preterm birth can be safely delayed, a retrospective observational analytical study demonstrated that expectant management is associated with improved neonatal outcomes compared with immediate delivery within 48 h [39]. The FIGO PremPrep-5 initiative provides a comprehensive framework aimed at improving preterm infant outcomes, including those born in the late preterm period. While interventions such as antenatal corticosteroids, magnesium sulfate for neuroprotection, delayed cord clamping, early breastfeeding, and immediate Kangaroo Care have demonstrated efficacy in reducing neonatal morbidity and mortality, their specific impact on late preterm infants warrants further investigation [40]. The current body of literature remains inconclusive regarding the efficacy of certain treatments, though a prospective observational study highlighting that administering MgSO_4_ may enhance fetal neurological outcomes and reduce the risk of cerebral palsy but it does not appear to impact mortality rates, unlike what was previously stated [41]. Additional research is needed to reinforce these findings and better understand the mechanisms involved. Unlike extremely preterm infants, late preterm infants often exhibit subtler neurological vulnerabilities that may not be immediately evident at birth but become more pronounced over time [40].

While some older studies suggested that the developmental gap between LPT and full-term infants may narrow over time, the differences often remain significant [42].

A comprehensive care bundle underscores the value of family support in neonatal care as demonstrated by the principles of family-centered care are integral to optimizing neurological outcomes in late preterm infants. PCPs should engage in shared decision-making with caregivers, providing reassurance when development is within the expected range while remaining vigilant for more subtle neurodevelopmental delays. Early referrals to specialized services, including speech therapy, occupational therapy, and neuropsychological assessments, can significantly improve long-term functional outcomes. Additionally, advocating for access to special education services and community-based resources ensures a holistic approach to supporting these children as they grow [43].

A collaborative team comprising healthcare professionals and the family should be responsible for the continuous monitoring of the child’s development to ensure progression appropriate to each developmental stage. Primary care pediatricians (PCPs) play a pivotal role in monitoring and addressing the neurodevelopmental risks associated with late preterm birth. These infants face an increased likelihood of cognitive, motor, and behavioral impairments, necessitating heightened surveillance and early intervention strategies. Developmental surveillance should be integrated into routine pediatric care, with particular attention to language acquisition, executive function, and social-emotional development. Given that many of these challenges manifest during school-age years, continuous monitoring beyond infancy is essential to ensure timely interventions and appropriate educational accommodations [43].

Given the increased risk of neurodevelopmental impairments, long-term follow-up care is crucial for preterm infants. Continuous monitoring can help identify issues early, allowing for timely interventions to improve quality of life and educational outcomes [9].

Despite advancements in neonatal care, challenges remain in ensuring consistent implementation of evidence-based interventions across different healthcare settings. In resource-limited environments, barriers such as limited access to neonatal intensive care units, trained healthcare professionals, and follow-up services may hinder optimal outcomes for late preterm infants. Addressing these disparities requires policy-driven initiatives aimed at improving the healthcare infrastructure and provider education.

Future studies should focus on refining intervention protocols and enhancing access to specialized care to bridge the gap in outcomes between late preterm and full-term infants.

## 5. Conclusions

Despite relatively low perinatal mortality, late preterm infants face a significantly heightened risk of neurodevelopmental impairments, including motor, cognitive, and behavioral challenges. Late preterm infants (34–36 weeks) demonstrated a 30% increased risk of experiencing at least one neurodevelopmental impairment by age 16 compared with their full-term peers, as this constitutes the focus of the review. Contributing factors such as early iatrogenic delivery, low birth weight, male sex, and socioeconomic disadvantage emphasize the necessity for individualized care and early screening. The role of nutrition, cerebral oxygenation, and emerging therapies—pharmacological and non-pharmacological—warrants further investigation, particularly given the subtle and often delayed manifestation of deficits in this population. Advances in neuroimaging and monitoring technologies offer promise for earlier identification of at-risk infants, but standardization in the definitions and assessment tools remains essential for meaningful comparisons across studies. A multidisciplinary, family-centered approach, integrated into primary care and supported by ongoing policy and research efforts, is key to optimizing outcomes. A key limitation of this review is that no study focused specifically on late preterm infants. Only one directly compared them with term infants, while others addressed broader preterm groups or lacked clear GA definitions. Relevant data on late preterm infants were selectively extracted for analysis. Future research should aim to refine and expand access to targeted interventions, with a continued focus on long-term follow-up to support these vulnerable infants throughout childhood and beyond.

## Figures and Tables

**Table 1:** Summary of the characteristics of included studies.

Authors	Title	Year	Study Type	Country	Measurements	Findings
Wolfsberger et al. [23]	Cerebral oxygenation immediately after birth and long-term outcome in preterm neonates—a retrospective analysis.	2023	Retrospective cohort study	Austria	Population, crSO_2_, cFTOE, SpO_2_, HR	Preterm neonates with adverse outcomes had beside lower GA and also a lower crSO_2_, SpO_2_, and HR
Gonzalez-Moreira et al. [19]	Prevention of neurological sequelae in preterm infants	2023	Retrospective cohort study	Mexico	Population, MRI evaluation, follow-up at 3 years, MDI, PDI, Katona’s neurohabilitation therapy	Significantly better outcomes at 3 years old compared with no treatment; the presence of sepsis and the volumes of the corpus callosum and lateral ventricles at 3–4 months were significant predictors of developmental outcomes at 3 years
Bobba et al. [20]	Early brain microstructural development among preterm infants requiring caesarean section versus those delivered vaginally	2023	Retrospective cohort study	USA	Population, delivery type, GA at birth/scan, MRI evaluation, WM maturation, 5 min APGAR, birth weight Z-score, maternal pre-eclampsia, chorioamnionitis	Preterm infants delivered by C-section show delayed white matter maturation, especially in the corpus callosum and internal capsule, independent of other factors
Paulsen et al. [7]	Early neurological and motor function in infants born moderate to late preterm or small for gestational age at term: a prospective cohort study	2023	Prospective cohort study	Norway	Population, neurological function, motor performance (HINE, GMA, TIMP, AIMS)	Preterm infants scored lower in neurological and motor assessments (HINE, GMA, TIMP, AIMS) compared with term AGA infants, indicating higher risks of developmental delays
Côté-Corriveau et al. [15]	Associations between neurological examination at term-equivalent age and cerebral hemodynamics and oxygen metabolism in infants born preterm	2023	Prospective cohort study	Canada	Population, FDNIRS, DCS, CBFi, SpO_2_, OEF, CMRO2i	FDNIRS-DCS parameters were not linked to neurological exams at TEA, greater CBFi and CMRO2i increases from birth to TEA, correlated with higher GA
Ericson et al. [10]	Equally good neurological, growth, and health outcomes up to 6 years of age in moderately preterm infants who received exclusive vs. fortified breast milk—a longitudinal cohort study	2023	Longitudinal cohort study	Sweden	Neurological development, growth parameters, health outcomes	No significant differences in neurological, growth, or health outcomes were found between preterm infants fed exclusive vs. fortified breast milk up to six years of age
Toma et al. [12]	Correlations between head ultrasounds performed at term-equivalent age in premature neonates and general movements neurologic examination patterns	2024	Pilot study	Romania	Population, TEA evaluation, GM, head ultrasound measurements	The CS movement pattern was associated with larger ventricular index, increased lateral ventricle measurements, and reduced basal ganglia width; the PR pattern showed an increased sinocortical width and reduced pons diameter
Triggs et al. [6]	The influence of birthweight on mortality and severe neonatal morbidity in late preterm and term infants: an Australian cohort study.	2024	Retrospective cohort study	Australia	Population, birthweight centile, outcomes (stillbirth, neonatal mortality, severe neurological morbidity, no adverse outcome)	Low birthweight increases stillbirth risk and neonatal mortality, while high birthweight increases severe neurological or other severe morbidity
Snir et al. [27]	Long-term neurological outcomes of offspring misdiagnosed with fetal growth restriction	2024	Retrospective cohort study	Israel	Population, misdiagnosis rate	Falsely diagnosed infants had higher risks of movement disorders, cerebral palsy, and developmental disorders
Mitha et al. [9]	Neurological development in children born moderately or late preterm: national cohort study	2024	Retrospective cohort study	Sweden	Population, follow-up from birth until the date of first diagnosis of the neurodevelopmental outcome, death, emigration, 16th birthday/31 December 2019	Late preterm have higher risks of adverse neurodevelopmental outcomes (any impairment; motor, cognitive, epileptic, visual, and hearing impairments, and severe or major neurodevelopmental impairment)
Zhang et al. [17]	Assessment of brain structure and volume reveals neurodevelopmental abnormalities in preterm infants with low-grade intraventricular hemorrhage	2024	Retrospective cohort study	China	Population, TEA evaluation, NBNA, MRI measurements	Preterm infants with low-grade IVH showed lower FA and MK values in the internal capsule and corpus callosum, changes linked to poorer neurodevelopmental outcomes at 40 weeks of corrected age
Toma et al. [14]	Cerebral ultrasound at term-equivalent age: Correlations with neuro-motor outcomes at 12–24 months corrected age	2024	Retrospective cohort study	Romania	Population, head ultrasound measurements, Amiel Tison neurologic examination	At 12 months, larger ventricular midbody and smaller basal ganglia were linked to abnormal motor outcomes; at 24 months, worse outcomes correlated with ventricular midbody >10.33 mm, smaller basal ganglia, reduced cortical depth, and immature gyral maturation
Lubián-Gutiérrez et al. [18]	Corpus callosum long-term biometry in very preterm children related to cognitive and motor outcomes	2024	Prospective cohort study	Spain	Population, MRI evaluation, CC, WPPSI-V, MABC-2	Children with smaller CC sizes had lower cognitive scores (FSIQ < 85) and motor function deficits (MABC-2 < 15th percentile)
Assar and Nouran Nasef [11]	Outcome of neonatal hyperbilirubinemia and its effect on neurological system in full term and preterm baby	2024	Cross sectional cohort study	Egypt	Population, clinical parameters, BIND score, follow-up at 3 months	Higher adverse outcomes (62.2%), mortality (32.4%), and neurological issues (29.7%); BIND score >5 predicted risk with 90.5% sensitivity
Toma et al. [8]	Early intervention guided by the general movements examination at term corrected age—short term outcomes	2024	Pilot study	Romania	Population, GMA, MOS-R	Early intervention improved outcomes in premature infants with cramped–synchronized GM patterns

## Abbreviations

The following abbreviations are used in this manuscript.

AGA	Appropriate for gestational age
AIMS	Alberta Infant Motor Scale
ASQ-2	Ages and Stages Questionnaires second Edition
BIND	Bilirubin-induced neurologic dysfunction
BPD	Bronchopulmonary dysplasia
BPF	Brain parenchymal fraction
BSID II/III	Bayley Scales of Infant and Toddler Development Second/Third Edition
CBFi	Index of microvascular cerebral blood flow
CC	Corpus callosum
cFTOE	Cerebral fractional tissue oxygen extraction
CMRO2i	Index of cerebral oxygen metabolism
CP	Cerebral palsy
crSO_2_	Cerebral regional oxygen saturation
CS	Cramped–synchronized
DCS	Diffuse correlation spectroscopy
DD/MR	Developmental delay/mental retardation
EUGR	Extrauterine growth restriction
FA	Fractional anisotropy
FDNIRS-DCS	Functional diffuse near-infrared spectroscopy and diffuse correlation spectroscopy
FGR	Fetal growth restriction
FiO_2_	Fraction of inspired oxygen
FSIQ	Full-Scale Intelligence Quotient
GA	Gestational age
GCC	Genu of the corpus callosum
GM	General movement
GMA	General Movements Assessment
HINE	Hammersmith Infant Neurological Examination
HR	Heart rate
IVH	Intraventricular hemorrhage
LISA/MIST	Less/minimally invasive surfactant administration/therapy
MABC-2	Movement Assessment Battery for Children-2
MDI	Mental Developmental Index
MK	Mean kurtosis
MOS-R	Motor optimality score—revised
MRI	Magnetic resonance imaging
NBNA	Neonatal Behavioral Neurological Assessment
NDI	Neurodevelopmental impairment
NICU	Neonatal intensive care unit
OEF	Cerebral oxygen extraction fraction
PCP	Primary care pediatrician
PDI	Psychomotor Developmental Index
PR	Poor repertoire
PPROM	Pre-labor preterm rupture of membranes
PVL	Periventricular leukomalacia
RBC	Red blood cell
rhEPO	Recombinant human erythropoietin
RK	Radial kurtosis
SpO_2_	Peripheral arterial oxygen saturation
TEA	Term-equivalent age
TH	Thalamus
TIMP	Test of Infant Motor Performance
WBCs	White blood cells
WM	White matter
WMI	White matter injury
WPPSI-V	Wechsler Intelligence Scale for Children-V
